# The impact of social and psychological support on patients with varying degrees of severity of chronic obstructive pulmonary disease

**DOI:** 10.3389/fmed.2025.1659691

**Published:** 2025-10-09

**Authors:** Sanhua Lian, Chunping Shi, Fengyu Chen, Zhixing Zhu, Xihua Lian

**Affiliations:** ^1^Department of Cardiothoracic Surgery, 910th Hospital of the People's Liberation Army Joint Logistics and Security Forces, Quanzhou, China; ^2^Department of Pulmonary and Critical Care Medicine, Respirology Medicine Centre of Fujian Province, Second Affiliated Hospital of Fujian Medical University, Quanzhou, China; ^3^Department of Ultrasound Medicine, Second Affiliated Hospital of Fujian Medical University, Quanzhou, China

**Keywords:** chronic obstructive pulmonary disease, social support, psychological support, pulmonary function, quality of life

## Abstract

**Objective:**

To investigate the effects of social support and psychological interventions on COPD patients across varying severity levels.

**Methods:**

This prospective, randomized controlled study included 172 COPD patients from two hospitals between January 2021 and June 2024. Finally, 132 participants were enrolled and randomly assigned to an intervention group (*n* = 66), receiving standard medical treatment plus systematic social and psychological support, or a control group (*n* = 66), receiving standard treatment and routine health education. The intervention lasted for 12 months. Primary outcomes, including quality of life (SGRQ), mental health (HADS), exercise capacity (6MWT), dyspnea (MRC scale), and pulmonary function (FEV1, FVC, FEV1/FVC), were assessed before and after the intervention.

**Results:**

No significant baseline differences were observed. After 12 months, the intervention group showed significant improvements in SGRQ, HADS, and MRC scores (*p <* 0.05), 6MWT distance, and pulmonary function (*p <* 0.05). Mild and moderate COPD patients in the intervention group showed significant improvements in all outcomes compared to baseline and the control group (*p <* 0.05). Severe and very severe patients showed improvements in SGRQ and HADS scores (*p <* 0.05), but no changes in 6MWT or pulmonary function (*p >* 0.05).

**Conclusion:**

Structured social and psychological interventions significantly improve quality of life, mental health, exercise capacity, and pulmonary function in mild to moderate COPD patients, but with limited effects on severe and very severe patients.

## 1 Introduction

Chronic obstructive pulmonary disease (COPD) is a progressive respiratory condition characterized by persistent airflow limitation. Its prevalence and mortality have been increasing significantly with global population aging, posing a serious burden on patients’ quality of life as well as on families and healthcare systems (1, 2). In addition to physiological symptoms, COPD is frequently associated with psychosocial issues such as social isolation, anxiety, and depression, which in turn negatively impact disease prognosis and increase the risk of acute exacerbations (3–7). Consequently, there is growing interest in incorporating social and psychological support as integral components of comprehensive COPD management strategies (1, 8).

An increasing body of evidence supports the efficacy of social and psychological interventions in improving outcomes for COPD patients (6, 9–11). A Dutch study reported that patients with inadequate social support experienced more severe symptoms, greater dependency on care, and higher levels of depression than those with adequate support (6). Liu et al. (9) further suggested that enhancing social support could alleviate anxiety and depression in patients with acute exacerbations, highlighting the need for caregivers and healthcare providers to prioritize psychosocial well-being. Other studies have demonstrated that structural social support—such as living with others or having a caregiver—is associated with higher physical activity and better adherence to pulmonary rehabilitation programs in adults with COPD, emphasizing the critical role of the social environment in promoting successful self-management (10). Turnier et al. (11) found that greater social support correlated with better clinical outcomes, including improved quality of life, reduced respiratory symptoms, and enhanced functional status.

However, most of the current literature has focused primarily on patients with mild to moderate COPD. Research involving patients with severe or very severe disease remains limited. Moreover, studies that do include a broad spectrum of disease severity often lack stratified analyses, making it unclear how patients at different stages respond to psychosocial interventions.

To address these gaps, the present randomized controlled trial aimed to evaluate the effects of structured social and psychological support on quality of life, mental health, physical activity, and pulmonary function in COPD patients across all severity levels. By comparing changes in St. George’s Respiratory Questionnaire (SGRQ), Hospital Anxiety and Depression Scale (HADS), 6-Minute Walk Test (6MWT), Medical Research Council (MRC), and lung function before and after a 12-month intervention, this study provides differentiated insights into the efficacy of psychosocial support among patients with mild, moderate, severe, and very severe COPD. The findings are intended to inform stage-specific, multidimensional intervention strategies in clinical practice.

## 2 Methods

### 2.1 Study participants

**Figure 1:**
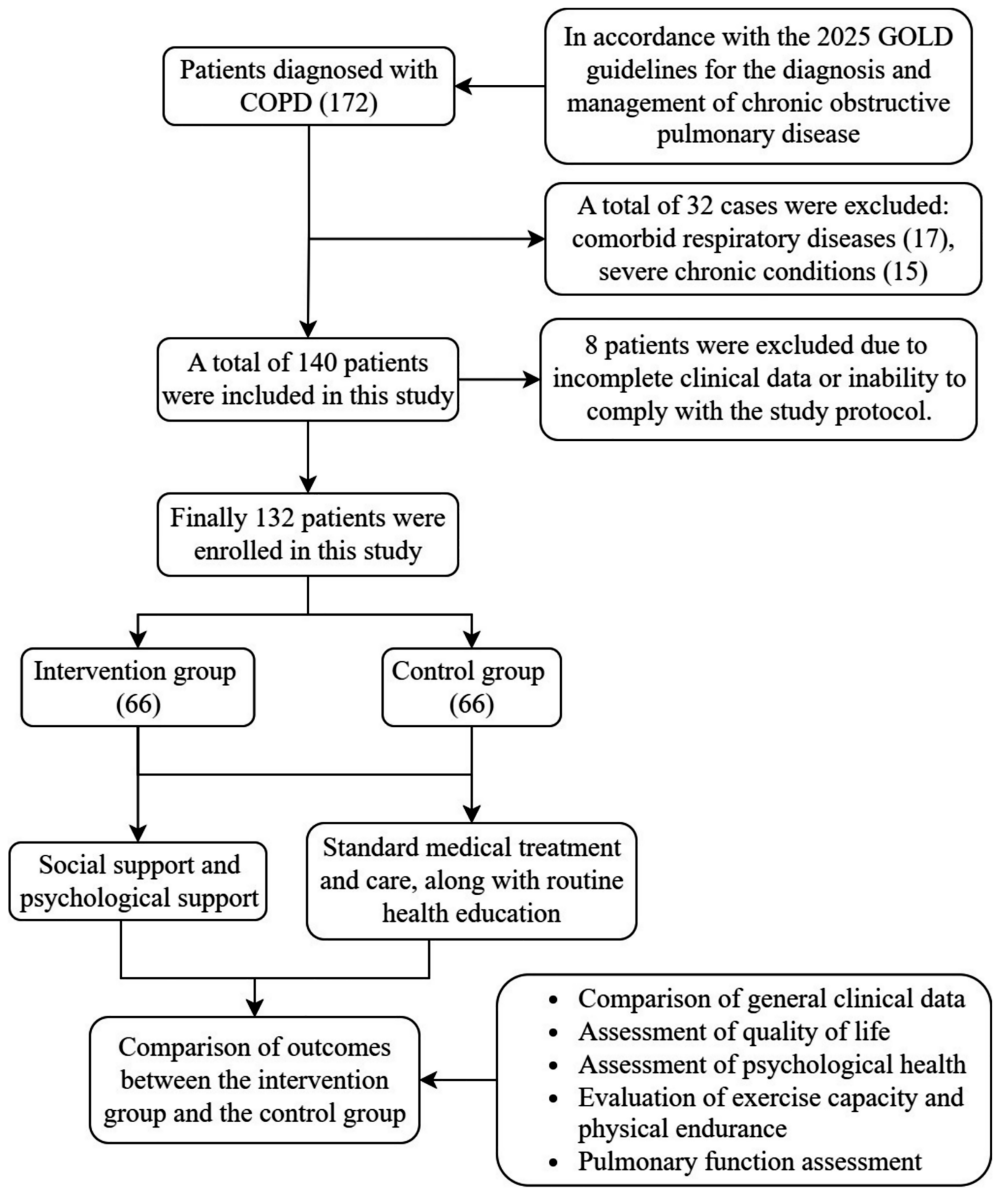
Flowchart of patient enrolment in this study. COPD, chronic obstructive pulmonary disease; GOLD, Global Initiative for Chronic Obstructive Lung Disease.

A total of 172 patients with varying degrees of COPD were consecutively recruited between January 2021 and June 2024 from two hospitals. All patients met the diagnostic criteria outlined in the 2025 Global Initiative for Chronic Obstructive Lung Disease (GOLD) guidelines (12). Of the 172 patients screened, 32 were excluded prior to randomization due to comorbid respiratory diseases (*n* = 17) or other chronic conditions (*n* = 15). The remaining 140 patients were randomized to the intervention group or the control group. After randomization, 8 patients were excluded because of incomplete clinical data or inability to comply with study procedures. Finally, 132 patients were included in the analysis, with 66 in the intervention group and 66 in the control group (Figure 1).

Inclusion criteria were as follows: (1) a confirmed diagnosis of COPD with complete radiological and pulmonary function data to determine disease severity; (2) the ability to understand and comply with study protocols and complete required questionnaires; and (3) age ≥18 years. Exclusion criteria included: (1) coexisting respiratory conditions such as bronchiectasis or asthma; (2) serious comorbid chronic diseases, such as malignancies or diabetic nephropathy; (3) significant psychiatric disorders, cognitive impairment, or inability to cooperate with study procedures; and (4) incomplete clinical data or inability to complete follow-up for any reason.

Eligible patients were randomly allocated to one of two groups using a computer-generated random number table to minimize selection bias. The intervention group received standard medical treatment in addition to structured social and psychological support, whereas the control group received standard medical care and routine health education without any additional psychosocial interventions. Randomization and group assignments were conducted by independent researchers to maintain methodological rigor.

The study protocol was approved by the Ethics Committee of 910th Hospital of the People’s Liberation Army Joint Logistics and Security Forces (NO. 2024-165) and the Ethics Committee of Second Affiliated Hospital of Fujian Medical University (NO. 2025-284), and conducted in accordance with the Declaration of Helsinki. All participants provided written informed consent prior to enrollment.

The sample size was determined based on primary outcomes including quality of life (SGRQ score), psychological health (HADS score), and pulmonary function (FEV1, FVC, and 6MWT). The expected effect size between the intervention and control groups was assumed to be moderate (Cohen’s d = 0.5), with a significance level of 0.05 and a statistical power of 80%. A 15% dropout rate was accounted for, and sample size calculations were based on comparing two independent means between groups. However, no participants were lost to follow-up after randomization. The final required sample size was 66 participants per group, totaling 132 patients, which was sufficient to detect significant differences in the primary outcomes.

### 2.2 Group allocation and intervention measures

*Control group*: Patients in the control group received standard medical treatment and routine nursing care, including general health education related to COPD.

*Intervention group*: In addition to receiving the same standard treatment and nursing care as the control group, patients in the intervention group were provided with structured social support and individualized psychological health interventions over a 12-month period (Supplementary material).

#### 2.2.1 Social support measures

1. *Educational and informational support*: Weekly 45-min health education sessions tailored to COPD patients were conducted, accompanied by the distribution of relevant educational materials to enhance patients’ disease knowledge and self-management skills.
2. *Emotional support*: Peer support groups were established, meeting fortnightly for 60 min to facilitate mutual experience sharing and emotional expression. Additionally, regular phone calls or text messages from trained volunteers were arranged to reduce patients’ sense of isolation and foster psychological connectedness.
3. *Practical assistance*: Practical daily-life support was offered, including help with managing medical appointments and navigating healthcare services. Patients were linked to a broader social service network comprising social workers and volunteer organizations.
4. *Social interaction and activities*: Monthly COPD rehabilitation events were organized, including light exercise or pulmonary rehabilitation activities to strengthen social bonds and promote participation in meaningful social activities, thereby enhancing overall quality of life and sense of self-worth.

#### 2.2.2 Individualized psychological support

1. *Psychological counseling*: Professional one-on-one psychological counseling sessions were conducted monthly (30–45 min each), focusing on emotional regulation, coping strategies, and family dynamics.
2. *Cognitive behavioral therapy*: A combination of group sessions and individual follow-ups was provided twice monthly to help patients identify and restructure maladaptive beliefs and build adaptive coping strategies such as relaxation techniques, mindfulness, and emotional self-regulation.
3. *Self-management support*: Patients received structured training in respiratory regulation techniques, delivered in weekly sessions and encouraged for daily self-practice. Stress management strategies, such as progressive muscle relaxation, guided imagery, and mindfulness meditation, were also introduced. Individualized stress coping plans, including personal triggers and corresponding coping actions, were developed collaboratively.
4. *Caregiver support*: Family members were invited to quarterly psychoeducational workshops covering supportive communication, caregiver burden recognition, and coping skills. A family support hotline or counseling service was made available to assist caregivers in managing stress and enhancing the overall effectiveness of the support system.

### 2.3 Outcome measures

#### 2.3.1 Demographic and clinical characteristics

Basic information was collected through structured interviews and medical records, including gender, age, occupation, education level, place of residence, disease duration, primary COPD symptoms, and disease severity at the time of consultation.

#### 2.3.2 Quality of life assessment

The SGRQ was used to assess changes in quality of life among COPD patients. The SGRQ consists of three components:

1. *Symptom score*: Assesses the impact of respiratory symptoms (e.g., dyspnea, cough, sputum) on the patient’s well-being. Scores range from 0 to 100, with higher scores indicating greater symptom burden.
2. *Activity score*: Evaluates the degree of limitation in physical activities due to breathlessness. Scores range from 0 to 100, where higher scores denote greater activity limitation.
3. *Impact score*: Reflects the psychosocial impact of the disease, including effects on emotional well-being and social functioning. Scores range from 0 to 100, with higher scores suggesting more severe impairment in quality of life.

Each section is scored based on patient responses to specific items. The total SGRQ score is a weighted average of the three domains, calculated using the following formula:


$$\begin{array}{l} \text{Total Score}=[(\text{Symptom Score}\times 40)/100]+\\ {}[(\text{Activity Score}\times 30)/100]+\\ \left[(\text{Impact Score}\times 30)/100\right] \end{array}$$

Higher total scores indicate worse overall health-related quality of life.

#### 2.3.3 Psychological health assessment

The HADS was used to evaluate the psychological status of COPD patients. The HADS consists of two subscales: the Anxiety Subscale (HADS-A) and the Depression Subscale (HADS-D), each comprising 7 items. Patients were asked to rate their experiences over the past week using a 4-point scale ranging from 0 to 3, with higher scores indicating more severe symptoms. Total scores for each subscale range from 0 to 21. A score of 0–7 is considered normal, 8–10 suggests mild symptoms, 11–14 indicates moderate symptoms, and 15 or above signifies severe anxiety or depression. These scores help to preliminarily identify the level of psychological distress and the potential need for further mental health intervention.

#### 2.3.4 Assessment of exercise capacity and physical endurance

6MWT and MRC dyspnea scale measures were used to further assess the functional status of COPD patients.

##### 2.3.4.1 6-min walk test

This test was performed according to the guidelines of the American Thoracic Society (ATS). Patients were instructed to walk as far as possible along a flat, straight, 30-meter corridor within a six-minute period, without running. The total distance walked (in meters) was recorded. Dyspnea levels were also assessed before and after the test using the MRC dyspnea scale.

##### 2.3.4.2 MRC dyspnea scale

The MRC dyspnea scale is a validated and widely used tool to assess the degree of breathlessness experienced during daily physical activities in COPD patients. It is a 5-point scale where higher scores indicate more severe dyspnea. The levels are defined as follows:

- Grade 1: Breathless only with strenuous exercise.
- Grade 2: Shortness of breath when hurrying on the level or walking up a slight hill.
- Grade 3: Walks slower than people of the same age on the level or stops for breath while walking at own pace.
- Grade 4: Stops for breath after walking about 100 meters or after a few minutes on the level.
- Grade 5: Too breathless to leave the house or breathless when dressing or undressing.

Scoring was performed through structured interviews conducted by trained healthcare staff, helping to objectively monitor the impact of interventions on patients’ respiratory function.

#### 2.3.5 Pulmonary function assessment

All COPD patients underwent pulmonary function testing, which included measurement of forced vital capacity (FVC), forced expiratory volume in one second (FEV1), and the FEV1/FVC ratio. These parameters were used to evaluate the severity of airflow limitation in accordance with the Global Initiative for Chronic Obstructive Lung Disease (GOLD) criteria. Based on clinical symptoms and FEV1/FVC values, COPD severity was classified into four categories: mild, moderate, severe, and very severe (Table 1).

**Table 1:** Classification of COPD severity.

Severity	FEV1/FVC	FEV1 (% predicted)	FVC (% predicted)	Primary symptoms
Mild	<70%	≥80%	Typically near normal; often > 80%	Mild symptoms, minimal limitation in daily activities
Moderate	<70%	50–79%	60–80%	Worsening symptoms with noticeable limitation in daily activities; exertional dyspnea may occur
Severe	<70%	30–49%	50–60%	Significant impact on daily life; frequent exertional dyspnea
Very severe	<70%	<30%	<50%	Extremely severe symptoms; dyspnea even at rest

FEV1, forced expiratory volume in one second; FVC, forced vital capacity.

To minimize assessment bias, outcome assessors were blinded to group allocation. Researchers responsible for data collection, including administering questionnaires (SGRQ, HADS, MRC) and conducting 6MWT and spirometry, were not involved in patient recruitment, treatment allocation, or intervention delivery. Group assignments were managed by independent coordinators, and participants were instructed not to disclose their allocation to assessors during follow-up visits. Data entry and statistical analysis were conducted by separate team members who remained blinded to group allocation until the primary analysis was complete.

#### 2.3.6 Assessment timeline

*Baseline Assessment*: Upon enrollment, eligible participants underwent baseline evaluation, which included collection of general demographic and clinical data, assessments of health-related quality of life, psychological status, exercise capacity, and pulmonary function.

*Follow-up Assessment*: After the 12-month intervention period, both groups were re-evaluated. Follow-up assessments encompassed the same parameters as baseline, including quality of life, psychological health, physical endurance, and pulmonary function measurements.

### 2.4 Statistical analysis

All statistical analyses were performed using SPSS software. For continuous variables, data conforming to a normal distribution were presented as mean ± standard deviation (SD), while data that did not follow a normal distribution or were non-continuous were reported as median and interquartile range (IQR). The Kolmogorov–Smirnov test was used to assess normality, and Levene’s test was used to evaluate homogeneity of variance. For comparisons between the intervention and control groups: If data were continuous, normally distributed, and variances were equal, independent samples *t*-tests were used. If data were not normally distributed, had unequal variances, or were non-continuous, the non-parametric Mann–Whitney *U* test was applied. To control for potential baseline differences, an analysis of covariance (ANCOVA) was performed for all outcome variables. Categorical data were expressed as percentages. Comparisons of categorical variables between groups were conducted using the chi-square test or Fisher’s exact test, as appropriate. A *p*-value of <0.05 was considered statistically significant.

## 3 Results

### 3.1 Comparison of baseline characteristics between groups

A comparison of baseline characteristics between the intervention group and the control group revealed no statistically significant differences in terms of gender, age, occupation, educational level, place of residence, duration of illness, primary COPD symptoms, or disease severity at the time of admission. These findings indicate that the two groups were comparable at baseline (Table 2).

**Table 2:** Comparison of baseline clinical characteristics between the intervention and control groups.

Variable	Intervention group	Control group
Sex [*n* (%)]	Male: 30 (45.5%)	Male: 34 (51.5%)
	Female: 36 (54.5%)	Female: 32 (48.5%)
Age (years)	69.44 ± 11.90	70.86 ± 12.42
Occupation [*n* (%)]	Enterprise/Institution: 14 (21.1%)	Enterprise/Institution: 16 (24.2%)
	Worker: 13 (19.7%)	Worker: 13 (19.7%)
	Farmer: 24 (36.4%)	Farmer: 22 (33.3%)
	Freelancer: 15 (22.7%)	Freelancer: 15 (22.7%)
Education level [*n* (%)]	Illiterate: 10 (15.2%)	Illiterate: 8 (12.1%)
	Primary: 6 (9.1%)	Primary: 14 (21.2%)
	Junior High: 19 (28.8%)	Junior High: 15 (22.7%)
	Senior High: 16 (24.2%)	Senior High: 12 (18.2%)
	College or above: 15 (22.7%)	College or above: 17 (25.8%)
Residence [*n* (%)]	Urban: 36 (54.5%)	Urban: 29 (43.9%)
	Rural: 30 (45.5%)	Rural: 37 (56.1%)
Duration of illness [*n* (%)]	<5 years: 16 (24.2%)	<5 years: 11 (16.7%)
	5–10 years: 28 (42.4%)	5–10 years: 33 (50.0%)
	11–20 years: 19 (28.8%)	11–20 years: 17 (25.8%)
	>20 years: 3 (4.5%)	>20 years: 5 (7.6%)
Primary symptoms [*n* (%)]	Cough: 66 (100%)	Cough: 66 (100%)
	Sputum: 52 (78.8%)	Sputum: 58 (87.9%)
	Dyspnea: 15 (22.7%)	Dyspnea: 14 (21.1%)
	Chest tightness: 4 (6.1%)	Chest tightness: 4 (6.1%)
Disease severity at admission [*n* (%)]	Mild: 37 (56.1%)	Mild: 39 (57.6%)
	Moderate: 12 (18.2%)	Moderate: 11 (17.4%)
	Severe: 13 (19.7%)	Severe: 12 (18.9%)
	Very severe: 4 (6.1%)	Very severe: 4 (6.1%)

#### 3.1.1 Comparison of outcomes between groups

At baseline, there were no statistically significant differences between the intervention and control groups across all measured parameters. After 12 months of intervention, both groups demonstrated improvements in quality of life, psychological status, physical endurance, and pulmonary function compared to their respective baselines. However, the improvements were more pronounced in the intervention group. Specifically, the intervention group showed significantly lower scores on SGRQ, HADS, and MRC dyspnea scale compared to the control group (*p <* 0.05). In addition, the 6MWT distance was significantly greater in the intervention group (*p <* 0.05), and lung function indices including FEV1/FVC ratio, FEV1, and FVC were also significantly improved compared to the control group (*p <* 0.05). Importantly, when baseline values were adjusted for using ANCOVA, these between-group differences remained robust and highly significant across all outcome measures (*p* < 0.001), further confirming the superiority of the intervention (Tables 3, 4).

**Table 3:** Comparison of quality of life, psychological status, physical performance, and exercise capacity between the intervention and control groups before and after the intervention.

Group	SGRQ score		HADS score		6MWT		MRC score	
	Before	After	Before	After	Before	After	Before	After
Control group	22 (15, 53.5)	18.5 (12.75, 40.25)	12 (8, 20)	9.5 (6.75, 15)	433.43 ± 146.82	442.28 ± 150.88	1 (0, 2)	1 (0, 2)
Intervention group	22 (12, 51.25)	12.5 (7.75, 32)	12.5 (8, 22)	7 (5, 12)	426.8 ± 155.6	512.0 ± 208.5	1 (0, 2)	0 (0, 1)
t/*U* value	0.023	2.259	−0.303	2.853	−0.251	2.201	−0.532	2.751
*p* value	0.982	0.024	0.762	0.004	0.802	0.03	0.595	0.006
*F* value	43.889		59.752		204.383		29.326	
*p* value	<0.001		<0.001		<0.001		<0.001	

SGRQ, St. George’s respiratory questionnaire; HADS, hospital anxiety and depression scale; 6MWT, 6-min walk test; MRC, medical research council.

**Table 4:** Comparison of lung function between the intervention and control groups before and after the intervention.

Group	FEV1/FEV		FEV1		FVC	
	Before	After	Before	After	Before	After
Control group	0.56 ± 0.12	0.56 ± 0.13	1.45 ± 0.59	1.65 ± 0.62	2.58 ± 1.01	2.94 ± 1.01
Intervention group	0.56 ± 0.13	0.62 ± 0.17	1.42 ± 0.57	1.97 ± 0.82	2.54 ± 1.00	3.15 ± 1.27
t/U value	0.028	2.375	−0.295	2.476	−0.206	2.02
*p* value	0.978	0.019	0.769	0.015	0.837	0.045
*F* value	38.755		87.481		29.686	
*p* value	<0.001		<0.001		<0.001	

FEV1, forced expiratory volume in one second; FVC, forced vital capacity.

Additionally, we analyzed whether social and psychological support interventions affected the quality of life, mental health, physical activity, and pulmonary function in COPD patients with varying disease severity. After 12 months of intervention, we found that in patients with mild to moderate COPD, the SGRQ scores, HADS scores, and MRC scores in the intervention group were significantly lower than both their baseline levels and those of the control group post-intervention (*p <* 0.05). The 6MWT distances were significantly higher, and pulmonary function indicators (FEV1, FVC, and FEV1/FVC) showed marked improvement (*p <* 0.05). Among patients with severe COPD, post-intervention SGRQ and HADS scores in the intervention group were significantly reduced compared to both baseline and control group levels (*p <* 0.05); however, there were no significant changes in 6MWT, MRC scores, or pulmonary function indicators (*p >* 0.05). For patients with very severe COPD, although post-intervention SGRQ and HADS scores were significantly lower than baseline levels (*p <* 0.05), changes in 6MWT, MRC scores, and pulmonary function were not statistically significant, nor were there any differences compared to the control group post-intervention (*p >* 0.05) (Table 5).

**Table 5:** Comparison of outcome indicators in the intervention group by COPD severity at baseline and after 12 months.

Group	Indicator	Mild	Moderate	Severe	Very severe
Control group-after intervention	SGRQ score	9 (13, 17)	27 (32, 37)	45.25 (49, 52.75)	68.25 (78.5, 87.25)
	HADS score	5 (7, 9)	12 (14, 15)	15.5 (19.5, 22)	29.25 (30.5, 31)
	6MWT	547.4 ± 49.4	410.5 ± 22.8	239.9 ± 34.0	112.5 ± 18.0
	MRC score	0 (0, 1)	1 (1, 2)	1.25 (2, 3)	3.25 (4, 4)
	FEV1/FEV	0.60 ± 0.11	0.60 ± 0.05	0.48 ± 0.08	0.28 ± 0.08
	FEV1	2.06 ± 0.29	1.52 ± 0.33	0.89 ± 0.13	0.37 ± 0.06
	FVC	3.52 ± 0.76	2.57 ± 0.66	1.88 ± 0.43	1.39 ± 0.30
Intervention group-before intervention	SGRQ score	9 (15, 20)	31.5 (41, 42.75)	56.5 (60, 70)	82.75 (86, 88.5)
	HADS score	6.5 (9, 11)	15 (16, 18.75)	24 (25, 26.5)	34.5 (37, 39.5)
	6MWT	540.2 ± 44.4	409.3 ± 30.6	219.7 ± 53.0	103.5 ± 32.2
	MRC score	0 (0, 1)	1 (2, 2)	2 (3, 3)	3 (3.5, 4.0)
	FEV1/FEV	0.60 ± 0.10	0.60 ± 0.10	0.50 ± 0.12	0.28 ± 0.11
	FEV1	1.85 ± 0.22	1.23 ± 0.11	0.72 ± 0.15	0.29 ± 0.08
	FVC	3.2 ± 0.74	2.13 ± 0.50	1.46 ± 0.22	1.13 ± 0.36
Intervention group-after intervention	SGRQ score	6 (8, 11)^*#^	20 (25, 29)^*#^	32.5 (38, 42.5)^*#^	65.25 (70.5, 78)^*^
	HADS score	3.5 (5, 6.5)^*#^	7.25 (9, 12)^*#^	11 (12, 14)^*#^	30 (31, 33.5)^*^
	6MWT	676.1 ± 44.2^*#^	440.3 ± 34.1^*#^	235.9 ± 51.6	106.4 ± 31.9
	MRC score	0 (0, 0)^*#^	0 (0.5, 1)^*#^	1 (2, 2.5)^*^	3 (3.5, 4)
	FEV1/FEV	0.66 ± 0.13^*#^	0.71 ± 0.17^*#^	0.74 ± 0.40	0.31 ± 0.10
	FEV1	2.51 ± 0.38^*#^	2.08 ± 0.18^*#^	1.70 ± 1.00	0.31 ± 0.06
	FVC	3.92 ± 0.93^*#^	3.00 ± 0.77^*^	2.31 ± 0.44	1.23 ± 0.33

SGRQ, St. George’s respiratory questionnaire; HADS, hospital anxiety and depression scale; 6MWT, 6-min walk test; MRC, medical research council; FEV1, forced expiratory volume in one second; FVC, forced vital capacity; ^*^p < 0.05, compared with the intervention group before the intervention, ^#^*p* < 0.05, compared with the control group after the intervention.

## 4 Discussion

This randomized controlled trial investigated the role of social and psychological support in the treatment of patients with COPD. The findings demonstrated that, compared with the control group, patients in the intervention group who received standard medical care in combination with additional social and psychological support for 12 months exhibited significantly lower scores on both the St. George’s Respiratory Questionnaire (SGRQ) and the Hospital Anxiety and Depression Scale (HADS). These results suggest a marked alleviation of anxiety and depressive symptoms in the intervention group.

In addition, pulmonary function indicators—including the FEV1/FVC ratio, FEV1, and FVC—were significantly improved in the intervention group, indicating better respiratory outcomes compared to the control group. Subgroup analysis further revealed that the intervention had the most pronounced effect among patients with mild to moderate COPD, who showed significant improvements across all measures relative to their baseline values. Among patients with severe COPD, SGRQ and HADS scores in the intervention group were significantly reduced compared to both baseline and control group levels, with no significance in the remaining indicators. In contrast, patients with very severe COPD exhibited no significant improvements in SGRQ and HADS scores when compared to control group levels.

Importantly, subgroup analysis revealed that adding social and psychological support had the most significant benefits for patients with mild to moderate COPD. Compared to both their own baseline and the control group post-intervention, these patients showed marked improvements in quality of life, psychological well-being, and lung function. This aligns with existing evidence: pulmonary rehabilitation combined with education and social support can significantly enhance 6MWT distance, SGRQ score, and MRC score, and even yield short-term gains in FEV1 (13–15). The likely explanation is that patients at these stages still retain sufficient physiological reserve and self-management capacity. Targeted psychosocial support reduces anxiety and depression, which in turn encourages participation in exercise, social activity, and engagement with therapeutic interventions—leading to more pronounced physiological and psychological improvements (14, 16).

Patients with severe and very severe COPD showed improvements mainly in quality of life and mental health, as reflected by reduced SGRQ and HADS scores, while gains in exercise capacity and lung function were minimal. These limited effects are likely due to irreversible structural lung damage, advanced airflow limitation, and diminished baseline functional reserve, which constrain the potential for recovery (17, 18). In very severe cases, additional factors such as reduced exercise tolerance, complex psychological needs, and lower compliance may further blunt intervention effects. Nevertheless, improvements in psychological well-being can still enhance disease acceptance and life satisfaction. In contrast, patients with mild to moderate disease derived broader benefits, including pulmonary function and physical endurance, likely mediated by enhanced adherence, reduced distress, and greater engagement in breathing exercises and activity through structured social and psychological support. These mechanisms, by reducing dynamic hyperinflation and optimizing respiratory efficiency, provide a plausible explanation for the superior outcomes observed in the intervention group and underscore the importance of early implementation of comprehensive supportive strategies.

In summary, the impact of social and psychological support on COPD patients varies according to disease severity, with the most pronounced benefits observed in individuals with mild to moderate COPD. These findings underscore the importance of early intervention to maximize therapeutic outcomes. In contrast, patients with severe or very severe COPD may require a more comprehensive approach that integrates personalized medical therapies—such as domiciliary oxygen or tailored pulmonary rehabilitation—with advanced psychological support to achieve meaningful gains across functional, psychological, and clinical domains.

However, this study has several limitations. First, it was conducted at two centers, which may limit generalizability due to potential geographic and demographic biases. Second, the analysis was restricted to short-term outcomes after 12 months; therefore, the durability of these interventions over the long term remains uncertain. Third, the limited response observed in very severe COPD patients suggests a need for more precisely targeted interventional strategies in this subgroup. Future research should involve larger, more diverse populations, extend the follow-up period, incorporate biomarker assessments to elucidate mechanisms of action, and develop stratified intervention protocols tailored to each stage of COPD severity.

## 5 Conclusion

In conclusion, structured social and psychological support play a critical role in enhancing quality of life, mental health, and pulmonary function in COPD patients. They are especially effective in mild to moderate cases and should be considered essential components of integrated COPD care.

## Data Availability

The original contributions presented in the study are included in the article/Supplementary material, further inquiries can be directed to the corresponding authors.
