# Supplementary material for: The impact of social and psychological support on patients with varying degrees of severity of chronic obstructive pulmonary disease
Source: Front Med (Lausanne). 2025 Oct 9;12:1659691. doi: 10.3389/fmed.2025.1659691 (PMC12545121; doi:10.3389/fmed.2025.1659691)
Supplement: Supplementary file 1 [file Table_1.docx]

**Detailed description of social support and individualized psychological interventions**

**(1) Social support measures**

1. **Educational and informational support**

Weekly 45-minute health education sessions were delivered by trained nurses and respiratory specialists. These sessions covered COPD pathophysiology, symptom recognition, medication adherence, inhaler technique, smoking cessation strategies, nutritional advice, and the importance of regular physical activity. Patients also received evidence-based educational booklets and leaflets written in accessible language to reinforce learning at home and improve long-term self-management skills.

1. **Emotional support**

Biweekly peer support group meetings (60 minutes each) provided a structured environment for patients to share personal experiences, discuss challenges, and exchange coping strategies under the facilitation of a psychologist or trained nurse. In addition, trained volunteers maintained regular phone calls or text messaging with patients, offering ongoing encouragement and reducing feelings of loneliness. These communications aimed to promote psychological connectedness and create a sense of belonging.

1. **Practical assistance**

Patients were offered support in scheduling medical appointments, arranging transportation, and navigating healthcare services. Dedicated staff helped link participants with social workers and community-based volunteer organizations. These networks provided resources such as home visit assistance, medication reminders, and help with daily living activities, thereby reducing barriers to accessing healthcare and improving overall adherence to treatment regimens.

1. **Social interaction and activities**

Monthly COPD rehabilitation events were organized in a group setting. These activities included light aerobic exercise, stretching, and pulmonary rehabilitation exercises supervised by physiotherapists, adapted to patients’ functional capacities. Social activities, such as group discussions, recreational games, or community events, were also incorporated. These interventions promoted peer interaction, strengthened social bonds, and enhanced patients’ self-esteem and sense of purpose.

**(2) Individualized psychological support**

1. **Psychological counseling**

Monthly one-on-one counseling sessions (30–45 minutes) were provided by licensed psychologists. The sessions addressed emotional regulation, stress coping strategies, and communication within family systems. Patients were encouraged to explore their concerns in a safe environment and were guided in reframing maladaptive coping patterns into constructive ones.

1. **Cognitive behavioral therapy**

Structured cognitive behavioral therapy interventions were delivered twice monthly, combining small group sessions and individual follow-ups. The program aimed to help patients identify irrational or maladaptive beliefs, for example, “My condition will only get worse; nothing can help me”, and replace them with adaptive cognitions. Behavioral components included relaxation training, mindfulness practices, and emotional self-regulation strategies. Homework assignments such as thought diaries and relaxation practice logs were used to reinforce learning between sessions.

1. **Self-management support**

Patients participated in weekly training sessions focusing on respiratory regulation techniques such as pursed-lip breathing and diaphragmatic breathing, with encouragement to practice daily at home. Stress management modules introduced progressive muscle relaxation, guided imagery, and mindfulness meditation. Together with clinicians, each patient developed an individualized coping plan that included identification of personal stress triggers and practical strategies to address them, such as structured daily routines, activity pacing, or relaxation before sleep.

1. **Caregiver support**

Family caregivers attended quarterly psychoeducational workshops that covered supportive communication skills, caregiver burden recognition, and stress management techniques. These workshops emphasized the caregiver’s role in encouraging adherence to treatment, promoting positive health behaviors, and providing emotional reassurance. A dedicated family support hotline and optional counseling services were available for caregivers to address their psychological needs and reduce stress. This approach aimed to strengthen the family support system and improve overall treatment outcomes.
